# Supplementary material for: Intercropping with Potato-Onion Enhanced the Soil Microbial Diversity of Tomato
Source: Microorganisms. 2020 Jun 2;8(6):834. doi: 10.3390/microorganisms8060834 (PMC7357159; doi:10.3390/microorganisms8060834)
Supplement: Supplementary file 1 [file microorganisms-08-00834-s001.pdf]

Table S1. Relative abundances of the bacterial genera in monoculture (M) and intercropping (I) systems (average relative abundances >0.30% in at least one treatment).

Taxa	M	I	Taxa	M	I
**Subgroup_6_norank**	**2.91** [1]	**5.34**	Cellvibrio	0.30	0.51
**Arthrobacter**	**3.11**	**4.03**	Rhizobium	0.42	0.51
**Sphingobium**	**24.29**	**3.71**	Acidibacter	0.41	0.51
**Flavobacterium**	**5.48**	**2.96**	Steroidobacter	0.35	0.50
Massilia	1.97	2.55	**Microlunatus**	**0.23**	**0.50**
**Bacillus**	**1.60**	**2.53**	Noviherbaspirillum	0.39	0.49
**Pseudomonas**	**1.74**	**2.27**	Pseudoduganella	0.64	0.47
**Mitochondria_norank**	**0.45**	**2.02**	Pseudoxanthomonas	0.61	0.47
Rhizobacter	1.64	1.72	Chitinophagaceae_uncultured	0.46	0.46
**Lysobacter**	**2.37**	**1.68**	**Gaiella**	**0.28**	**0.45**
**Gemmatimonadaceae_uncultured**	**0.82**	**1.48**	**Actinobacteria_norank**	**0.25**	**0.44**
Streptomyces	1.34	1.48	Flavisolibacter	0.43	0.44
**Nitrosomonadaceae_uncultured**	**0.93**	**1.48**	Comamonadaceae_unclassified	0.50	0.43
**Variovorax**	**2.05**	**1.36**	Opitutus	0.36	0.43
Ramlibacter	1.66	1.34	Pseudolabrys	0.34	0.42
**Gemmatimonas**	**0.82**	**1.29**	Mesorhizobium	0.37	0.40
WD2101_soil_group_norank	0.85	1.11	**GR-WP33-30_norank**	**0.21**	**0.39**
Oxalobacteraceae_unclassified	1.07	1.10	**Pirellula**	**0.25**	**0.38**
RB41_norank	0.63	1.00	Blastococcus	0.27	0.38
**Anaerolineaceae_uncultured**	**0.58**	**0.91**	**Candidatus_Solibacter**	**0.19**	**0.37**
**Nitrospira**	**0.42**	**0.87**	Blastocatella	0.32	0.36
**SC-I-84_norank**	**0.68**	**0.85**	Sphingobacteriaceae_unclassified	0.50	0.36
**Gaiellales_uncultured**	**0.45**	**0.85**	Phenylobacterium	0.32	0.35
Sphingomonas	0.52	0.85	**Reyranella**	**0.20**	**0.35**
**Devosia**	**0.67**	**0.79**	Altererythrobacter	0.46	0.34
**Bryobacter**	**0.42**	**0.71**	**C0119_norank**	**0.21**	**0.34**
**Bacillales_unclassified**	**0.27**	**0.70**	**Dyadobacter**	**1.05**	**0.33**
**Bradyrhizobium**	**0.52**	**0.69**	Cytophagaceae_uncultured	0.32	0.33
**Subgroup_1_uncultured**	**0.40**	**0.68**	Ohtaekwangia	0.45	0.33
**Luteimonas**	**0.87**	**0.68**	Aeromicrobium	0.30	0.33
**Plantactinospora**	**0.41**	**0.65**	**Caulobacter**	**0.53**	**0.33**
**ABS-19_norank**	**0.31**	**0.61**	**Haliangium**	**0.15**	**0.32**
**Subgroup_7_norank**	**0.30**	**0.59**	Skermanella	0.18	0.30
Arenimonas	0.44	0.56	Armatimonadetes_norank	0.21	0.30
KD4-96_norank	0.27	0.56	Pseudorhodoferax	0.40	0.24
Xanthomonadaceae_unclassified	0.65	0.53	Xenophilus	0.40	0.23
Nocardioides	0.55	0.53	Chitinophaga	0.33	0.20
Methylotenera	0.56	0.52	Stenotrophomonas	0.34	0.15

[1] Values in bold are significant ($p < 0.05$) according to Student's t-test.

Table S2. Relative abundances of the fungal genera in monoculture (M) and intercropping (I) systems (average relative abundances >0.30% in at least one treatment).

Taxa	M	I	Taxa	M	I
Fusarium	15.68 [1]	8.22	Davidiella	0.41	0.30
Mortierella	11.41	11.61	Aspergillus	0.26	0.35
**Cladosporium**	**16.43**	**6.40**	Microdochium	0.15	0.42
Gibberella	11.21	9.35	Epicoccum	0.17	0.33
Alternaria	4.62	4.64	**Lasiosphaeriaceae_unidentified**	**0.56**	**0.70**
Chaetomium	2.98	3.88	Sordariales_unclassified	0.35	0.64
Arnium	1.84	2.45	Hypocreales_unclassified	0.48	0.41
Curvularia	1.38	2.62	Ascomycota_unclassified	0.24	0.60
Guehomyces	1.72	1.93	Pleosporales_unclassified	0.50	0.27
Schizothecium	1.73	1.87	Phoma	0.31	0.39
**Olpidium**	**0.29**	**2.78**	Zopfiella	0.36	0.22
Humicola	1.08	1.08	Thelebolales_unclassified	0.44	0.14
Penicillium	0.81	1.03	Myrmecridium	0.03	0.45
Acremonium	0.58	0.58	Lasiosphaeriaceae_unclassified	0.32	0.13
Chaetomidium	0.24	0.91	Apodus	0.11	0.33
**Cryptococcus**	**0.29**	**0.81**			

[1] Values in bold are significant ($p < 0.05$) according to Student's t-test.
